# An Unusual and Difficult to Detect Cause of Infection in Two Trauma Patients

**DOI:** 10.1093/cid/ciac748

**Published:** 2022-10-06

**Authors:** Kim Yeoh, Dilare Aikeremu, Benjamin Aw-Yeong, Monica A Slavin, Eloise Williams

**Affiliations:** Department of Microbiology, The Royal Melbourne Hospital, Melbourne, Victoria, Australia; Department of Infectious Diseases, University of Melbourne at the Peter Doherty Institute for Infection and Immunity . Melbourne, Victoria, Australia; Department of Microbiology, The Royal Melbourne Hospital, Melbourne, Victoria, Australia; Victorian Infectious Diseases Service, Royal Melbourne Hospital, Melbourne, Victoria, Australia; Department of Infectious Diseases, University of Melbourne at the Peter Doherty Institute for Infection and Immunity . Melbourne, Victoria, Australia; Department of Infectious Diseases, and National Centre for Infections in Cancer, Peter MacCallum Cancer Centre, Melbourne, Victoria, Australia; Victorian Infectious Diseases Service, Royal Melbourne Hospital, Melbourne, Victoria, Australia; Department of Microbiology, The Royal Melbourne Hospital, Melbourne, Victoria, Australia; Department of Infectious Diseases, University of Melbourne at the Peter Doherty Institute for Infection and Immunity . Melbourne, Victoria, Australia

**Question:**

**CASE 1**

A previously well 17 year-old male involved in a motor-vehicle accident sustained multiple injuries including left acetabular and displaced comminuted pelvic fractures, both requiring surgical fixation. Pudendal artery damage resulted in a left retroperitoneal, extraperitoneal, and preperitoneal hematoma. He received intensive supportive care, including empiric intravenous amoxicillin-clavulanate, a central venous catheter, and a urinary catheter.

On day 8, fevers developed and piperacillin-tazobactam and vancomycin were commenced. A computerized tomography (CT) scan of the chest, abdomen, and pelvis did not reveal an infective source. Fevers persisted, and he developed increasing left hip pain. A repeat CT scan on day 13 revealed a new collection around the left lateral pelvic wall, contiguous with the pelvic fixation and left hip joint (Figure 1). On day 16, an ultrasound guided aspirate of the pelvic collection was performed, and he underwent pelvic washout and debridement.

**Figure 1.**
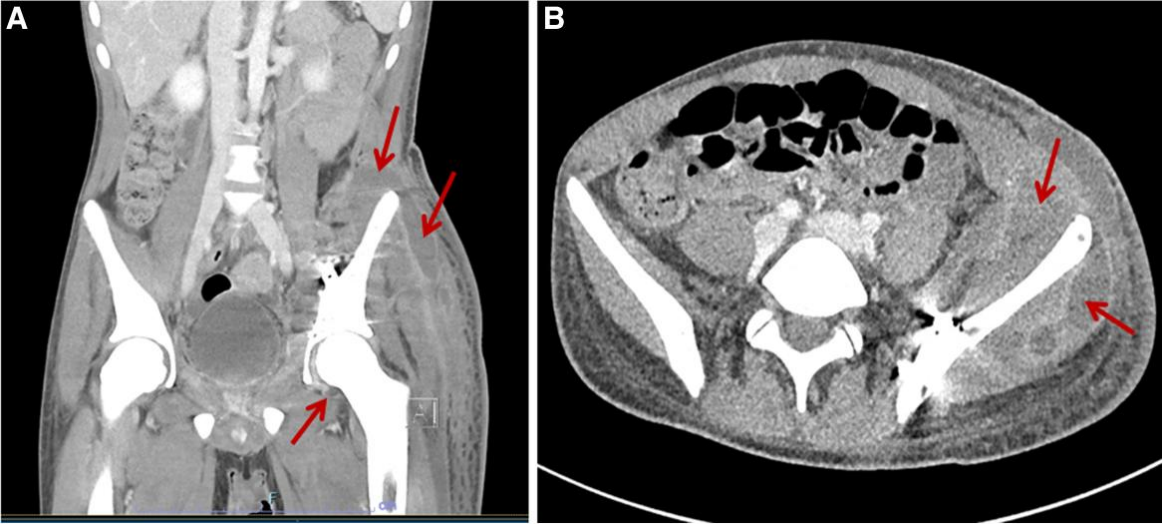
*Case 1*: CT abdomen and pelvic portal venous phase intravenous contrast enhanced CT scan. *A*, Coronal plane image with arrows pointing to fluid collections. *B*, Sagittal plane image with arrows pointing to fluid collections.

The pelvic aspirate had 37 000 leucocytes (40 × phase contrast). No organisms were seen on Gram stain. At day 2 of incubation on horse blood agar (HBA) (Thermofisher, Waltham, Massachusetts, USA) in anaerobic conditions at 35°C, tiny translucent colonies had grown (Figure 3*A*), which were identified using matrix-assisted laser desorption ionization time-of-flight mass-spectrometry (MALDI-TOF MS). A color change occurred with subculture onto specialized agar (Figure 3*B*). Examination under direct microscopy revealed 2 organisms with distinct colony morphologies (Figure 3*C*).

**CASE 2**

Several months later, a previously well 17 year-old woman involved in a motor-vehicle accident sustained injuries including a shattered right kidney with a large perinephric hematoma requiring a nephrouretectomy; a left ureteric transection requiring anastomosis and stenting; and D3/4 duodenal tear managed with resection via laparotomy. Intraoperatively, a large volume retroperitoneal urinoma was drained. She commenced empiric piperacillin-tazobactam.

Retroperitoneal fluid microscopy and Gram stain were unrevealing. Culture was negative for growth. After 8 days of piperacillin-tazobactam, she developed further fevers and rising inflammatory markers. Meropenem was commenced. A CT intravenous pyelogram (Figure 2) revealed ongoing urinary leak. Abdominal and pelvic CT revealed a complex collection.

**Figure 2.**
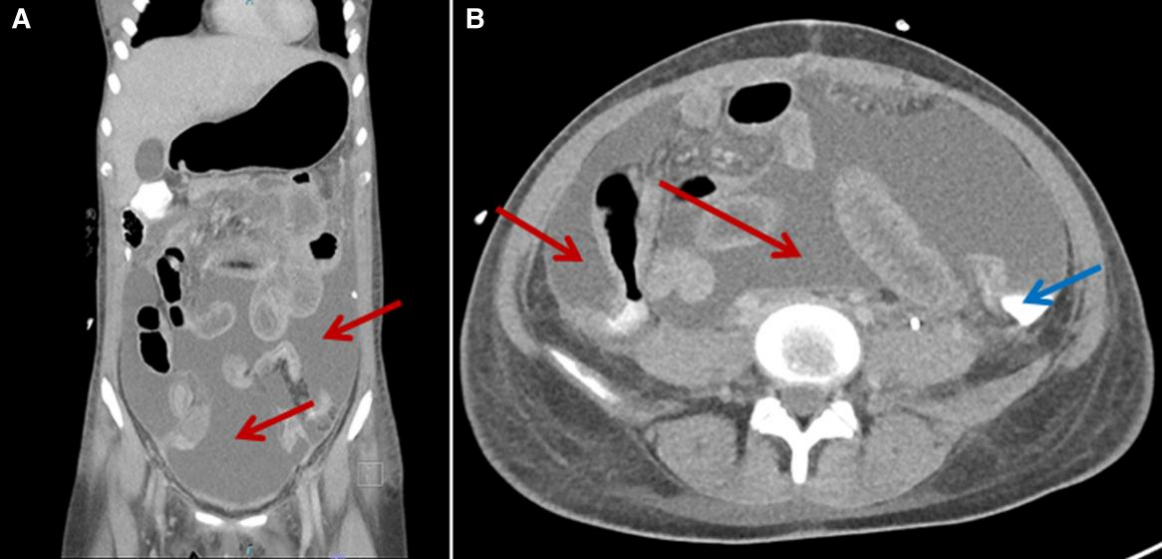
*Case 2*: CT abdomen and pelvis (intravenous pyelogram). *A*, Coronal plane image with arrows pointing to fluid collections. *B*, Sagittal plane image with red arrows pointing towards fluid collection and blue arrow pointing towards contrast leakage in the left perinephric space indicating left renal urinary leak from the pelviureteric junction.

This abdominal fluid was drained. After 4 days of incubation on HBA plates in anaerobic conditions at 35°C, tiny translucent colonies were revealed, identified using MALDI-TOF MS and confirmed with real-time polymerase chain reaction using the Anylpex II STI-7 assay (Seegene, Seoul, South Korea). After 2 days of subculture onto specialized agar, direct microscopy revealed 2 organisms with distinct colony morphologies identical to Case 1 (Figure 3*C*).

**Figure 3.**
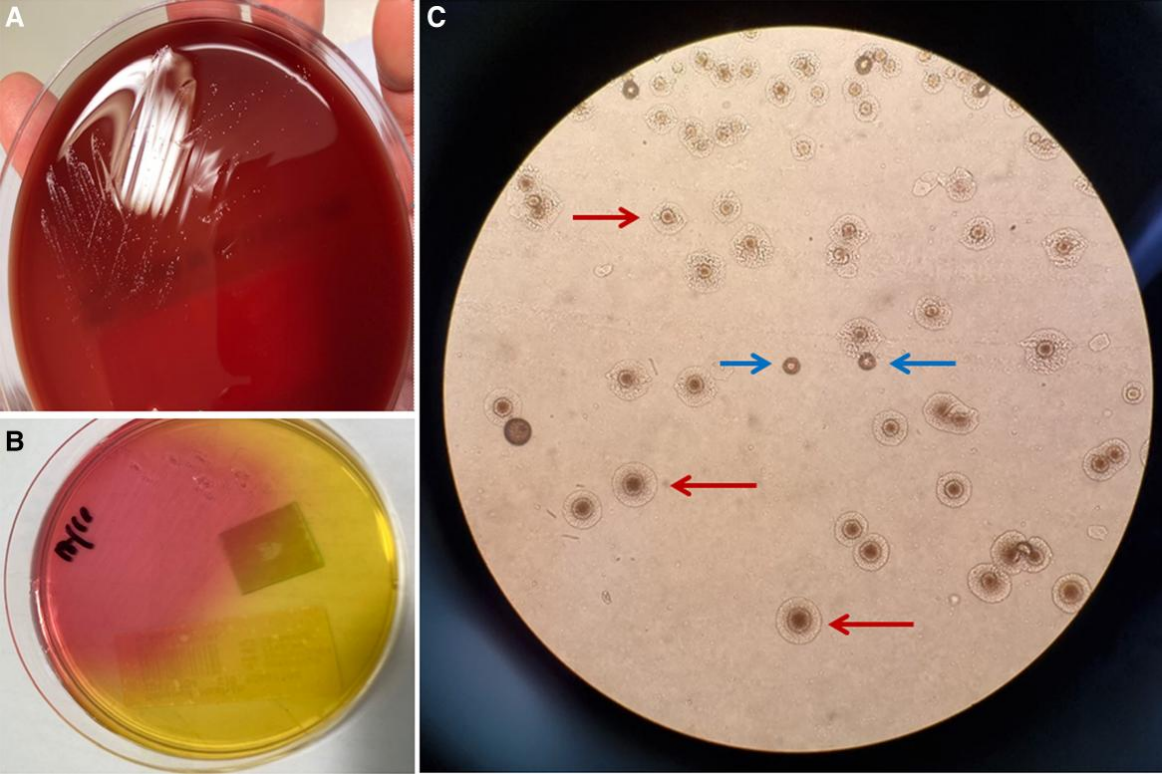
*A*, Tiny translucent white colonies on horse blood agar (Thermofisher, Waltham, USA) at 48 hours of incubation in anaerobic conditions at 35°C. *B*, Color change of A8 agar (Media Preparation Unit, University of Melbourne, Parkville, Australia) resulting from urease production in the presence of the CaCl_2_ indicator contained in the medium. *C*, Fluid from left pelvic collection. Colony morphologies identified on A8 agar after 48 hours of anaerobic incubation and viewed with a stereomicroscope (x40 magnification). Red arrows indicating an organism with a dense center and paler outer zone. Blue arrows indicating an organism with a brown granular appearance.

What is the diagnosis?

**Answer:**

*Mycoplasma hominis* and *Ureaplasma* species infections.

Case 1 had a *Mycoplasma hominis* and *Ureaplasma urealyticum* pelvic collection and metalware infection, and left hip septic arthritis (culture and polymerase chain reaction [PCR] positive). We propose that instrumentation of the genitourinary tract with a urinary catheter led to hematogenous metastatic seeding of these organisms. He was managed with doxycycline and ciprofloxacin, and his fevers subsided and pain improved.

Case 2 had a *M. hominis and Ureaplasma* species retroperitoneal urinoma likely secondary to the ongoing urinary leak. The patient was commenced on doxycycline and demonstrated clinical improvement.

## DISCUSSION

*Mycoplasma hominis* and *Ureaplasma* spp. belong to the Mollicutes class of bacteria. They lack a cell wall and therefore are unable to be visualized by Gram stain. They are normal commensal genitourinary tract flora in sexually active adults and can disseminate to other sites following mucosal disruption [1–6], most commonly in immunocompromised patients, particularly secondary to congenital immune defects, and also hematological malignancy and solid organ transplant [7–9].

The detection of Mollicutes in clinical specimens is challenging. It should be considered when initial microbiological testing (eg, Gram stain, routine culture) is unrevealing or if the patient does not improve on empiric antimicrobial therapy for more common pathogens [1, 10]. If these organisms are suspected, the specimen should be inoculated into broth culture media, sub-cultured onto A8 agar, and incubated for at least 48 hours under anaerobic conditions, both forming tiny pinpoint colonies. When viewed under a stereomicroscope, *M. hominis* colonies have a “fried egg” appearance with a denser center and paler outer zone, whereas *Ureaplasma* spp. colonies have a brown granular appearance. These miscroscopic findings are pathognomonic for these organisms without any legitimate differential diagnoses [8, 11]. Nucleic acid amplification tests (eg, PCR assay) can expedite pathogen identification [8, 12–14].

Mollicute septic arthritis is rare [1, 8]. Although *M. hominis* or *Ureaplasma* spp. single organism septic arthritis and periprosthetic joint infections have been reported in immunocompromised hosts [7, 15–21], there are very few case reports involving immunocompetent patients [5, 15, 20, 22–27]. To our knowledge, there is only 1 previous case of dual infection with *M. hominis* and *U. parvum* septic arthritis in an immunocompromised patient [28]. To the best of our knowledge, Case 1 is the first case of an immunocompetent patient developing dual Molliculite septic arthritis with metalware infection.

There are few cases of dual *Mycoplasma* and *Ureaplasma* non-genitourinary infection. In addition to Haller et al (1991) above [28], other authors have described dual infections in patients who underwent liver transplantation complicated by colonic perforation managed with ciprofloxacin; coronary artery bypass surgery with sternal wound infection treated with intravenous clindamycin and doxycycline, then oral doxycycline [29]; and mediastinitis, pleuritis, and pericarditis following cardiothoracic surgery in an immunocompromised patient, managed with doxycycline and clindamycin but succumbed to disseminated infection [30].

Understanding optimal antimicrobial selection is limited by lack of standardized methods and clinical breakpoints for antimicrobial susceptibility testing. Additionally, clinical data on the treatment of these organisms are limited to case reports and series, with limited data on clinical or microbiological response [1, 31–34]. Beta-lactam antibiotics and vancomycin are inactive because they target the cell wall. *Myoplasma* and *Ureaplasma* spp. are generally susceptible to agents that inhibit protein synthesis [35]. Fluoroquinolones, which are bactericidal against mycoplasmas should be considered for empiric treatment. Doxycycline should also be considered, although resistance may be increasing [1]. It is reasonable to use combination therapy (eg, moxifloxacin and doxycycline) [1, 34].

## CONCLUSION

Mollicutes should be considered as a cause of infection in patients sustaining disruption to genitourinary tract mucosa, or where there has been difficulty in isolating an organism or inadequate response to empiric antibiotics of more common organisms. There are limitations of susceptibility testing of these organisms. Empiric treatment of non-genital infections with fluroquinolones and tetracyclines should be considered.

## Notes

***Financial support.*** There were no sources of funding for this paper.
